# Quantitative proteomics reveals altered expression of extracellular matrix related proteins of human primary dermal fibroblasts in response to sulfated hyaluronan and collagen applied as artificial extracellular matrix

**DOI:** 10.1007/s10856-012-4760-x

**Published:** 2012-09-19

**Authors:** Stephan A. Müller, Anja van der Smissen, Margarete von Feilitzsch, Ulf Anderegg, Stefan Kalkhof, Martin von Bergen

**Affiliations:** 1 Department of Proteomics, UFZ, Helmholtz-Centre for Environmental Research Leipzig, 04318 Leipzig, Germany; 2 Department of Dermatology Venerology and Allergology, Leipzig University, 04103 Leipzig, Germany; 3 Department of Metabolomics, UFZ, Helmholtz-Centre for Environmental Research Leipzig, 04318 Leipzig, Germany; 4 Collaborative Research Center (SFB-TR67), Matrixengineering, Leipzig, Germany

## Abstract

Fibroblasts are the main matrix producing cells of the dermis and are also strongly regulated by their matrix environment which can be used to improve and guide skin wound healing processes. Here, we systematically investigated the molecular effects on primary dermal fibroblasts in response to high-sulfated hyaluronan [HA] (hsHA) by quantitative proteomics. The comparison of non- and high-sulfated HA revealed regulation of 84 of more than 1,200 quantified proteins. Based on gene enrichment we found that sulfation of HA alters extracellular matrix remodeling. The collagen degrading enzymes cathepsin K, matrix metalloproteinases-2 and -14 were found to be down-regulated on hsHA. Additionally protein expression of thrombospondin-1, decorin, collagen types I and XII were reduced, whereas the expression of trophoblast glycoprotein and collagen type VI were slightly increased. This study demonstrates that global proteomics provides a valuable tool for revealing proteins involved in molecular effects of growth substrates for further material optimization.

**Electronic supplementary material:**

The online version of this article (doi:10.1007/s10856-012-4760-x) contains supplementary material, which is available to authorized users.

## Introduction

The skin is the largest organ of the human body. It has many essential functions like body temperature regulation, oxygen uptake, pathogen defense and fluid loss prevention. Thus dermal wounds can cause severe health problems by the restriction of these functions. The therapeutic band width of skin wound treatment includes dressing with autografts, allografts, xenografts or tissue-engineered skin substitutes (TESS). TESS have been proven to be a good alternative to conventional treatment by grafting of skin wounds [1]. Clinical products from different companies are extensively reviewed by Eisenbud et al. [2] and Damanhuri et al. [3], while Metcalfe and Ferguson [4] have reviewed developments of bioengineered artificial skin. The usage of cell-free scaffolds as matrix supports for self-regeneration of skin is an alternative to skin biopsies and dermal cell culturing. Especially cell-free scaffolds based on biodegradable substances like polylactides, collagens and/or glycosaminoglycans (GAGs) which mimic the extracellular matrix (ECM) are good alternatives to conventional skin grafting [5–8].

A promising approach for the development of new artificial ECMs (aECMs) for wound healing of skin tissue is the integration of chemically modified natural ECM components. In particular sulfated GAG have been supposed to improve wound healing of skin tissue by the interaction of negatively charged sulfate groups with cytokines, growth factors and dermal cells [9, 10].

Sulfated derivatives of GAGs mimic the behavior of heparin, the most biological active natural GAG compound which plays an important role in wound healing [11]. Heparin interacts with a huge variety of different proteins, like growth factors FGFs (fibroblast growth factors)-1, -2 and -7 [12] or cytokines such as platelet factor 4 [13], interleukin 8 (IL-8) [10, 14] or interferon gamma [15]. Heparin further binds to adhesion proteins like selectins [16], the heparin-binding growth associated molecule [13] and fibronectin [17]. Protein binding to heparin promotes different functions like protection from proteolysis (i.e. FGFs-1, -2 and -7) [12, 18] or modification of biological activity shown for transforming growth factor β1 (TGF-β1) [13]. Thus heparin and other sulfated GAG have an influence on key processes of wound healing like inflammation, cell proliferation or cell–matrix interactions [13]. Most interactions between sulfated GAG and proteins are governed by negatively charged sulfate groups which form ionic bonds with basic amino acid residues [10, 12, 13, 15].

Hence, cell studies with sulfated GAG can provide valuable information for the engineering of new skin substitutes. We have chosen hyaluronan (HA) to investigate the effect of chemical sulfation. HA is the most suited GAG for this study since naturally HA does not contain sulfate groups. It has a regular sequence of alternating units of *N*-acetylglucosamine and glucuronic acid and is not covalently linked to proteins. Additionally, HA can be chemically modified without loss of structure [11]. Since our research focus is on acquiring knowledge about the influence of synthetized aECMs for improved wound healing of skin tissue we have chosen dermal fibroblasts (dFbs) as model cells for investigation of our modified aECM. They are crucial for wound healing of skin tissue and strongly regulated by their surrounding ECM [19]. The previous work of van der Smissen et al. [20] showed that sulfated GAGs improved initial cell adhesion and proliferation of dFbs in a sulfation dependent manner. By testing a few selected mRNA of involved key proteins the expression levels of collagen type I α chain, HA synthase 2 and matrix metalloproteinase-1 (MMP-1) were found to be significantly reduced on high-sulfated GAGs, whereas low-sulfated GAG derivatives only slightly changed the mRNA expression of these components.

On the basis of these data [20], the influence of HA sulfation on the expression of other proteins by a non-targeted approach is of great interest since this will allow detecting so far unrecognized signaling pathways in response to the tested biomaterials. We analyzed the influence of aECMs consisting of collagen type I mixed with HA or its high-sulfated derivative (hsHA) on protein level. For that reason, we have chosen stable isotope labeling by amino acids in cell culture (SILAC) which is a well-established method enabling accurate relative quantification of thousands of proteins in an untargeted approach [21, 22]. As long as primary cells can be cultivated for a sufficient time to obtain quantitative isotope labeling, SILAC provides superior protein coverage and better quantitative reproducibility in comparison to the usage of cells or organs from different individuals or label free quantification [23]. Especially relative quantification to a control of the same donor within one measurement reduces variability.

Global analyses provide a broader overview and higher protein coverage than targeted experiments. Computational analyzes of regulated proteins according to databases like PANTHER (Protein ANalysis THrough Evolutionary Relationships) [24] reveal protein cluster enriched according to their molecular functions and biological processes. Bioinformatics tools like DAVID [25] additionally calculate enrichment factors and determine statistical significance of these clusters.

While these approaches are limited to detecting known pathways the global approach also offers the chance to unravel so far unknown proteins or complexes that might also be pivotal to the process of interest. In order to extract this potential from the wealth of raw data gathered through omics approaches it is necessary to build up cell type and research specific databases. More specifically the effects of aECM on different cell types involved in wound healing should be summarized in a database allowing a focused comparison with future data.

A generally important aspect of global analysis is the assumption that the conditions do not cause an overall extreme stress to the cells, since then the effects would reflect all but not dominantly specifically mechanism about the subtle changes occurring during adaptation. The general effects can be monitored by the amount of overall changes and as a valid assumption the significantly (*P* < 0.05) changed proteins should not exceed 5–10 %.

In this study over 2,000 proteins were unambiguously identified and the gene enrichment process revealed that HA sulfation affects predominantly ECM remodeling by simultaneously down-regulation of ECM degenerating proteins like MMPs-2 and -14 as well as cathepsin K (catK). Additionally, other ECM proteins including decorin, thrombospondin-1 (TSP-1), and collagen types I, VI and XII are regulated. Beside this detailed information on coordinated ECM remodeling the summary of affected pathways and molecular functions allows to build a database for monitoring of aECM caused effects on fibroblasts.

## Materials and methods

### Sample preparation

The study was conducted according to Declaration of Helsinki Principles (1975) and was approved by the local ethics committee (065-2009).

Primary human dFbs from healthy breast skin were isolated as previously described [26] by dispase II (Roche Diagnostics GmbH, Mannheim, Germany) mediated removal of epidermal sheet and digestion of the dermal compartment with collagenase (Sigma-Aldrich Chemie GmbH, Steinheim, Germany). Cell suspension was passed through 70 μM filters (BD Biosciences, Bedford, MA, USA) to remove tissue debris. In total four biological replicates deriving from different donors were applied in this study.

Cells were cultured with Dulbecco’s Modified Eagle Medium (DMEM, Biochrom AG, Berlin, Germany) supplemented with 10 % fetal calf serum (FCS, Biochrom AG, Berlin, Germany) and 1 % penicillin/streptomycin (PAA Laboratories GmbH, Pasching, Austria) at 37 °C, 5 % CO_2_ until confluence. For experiments cells between passages 2–8 were used [20].

**Fig. 1:**
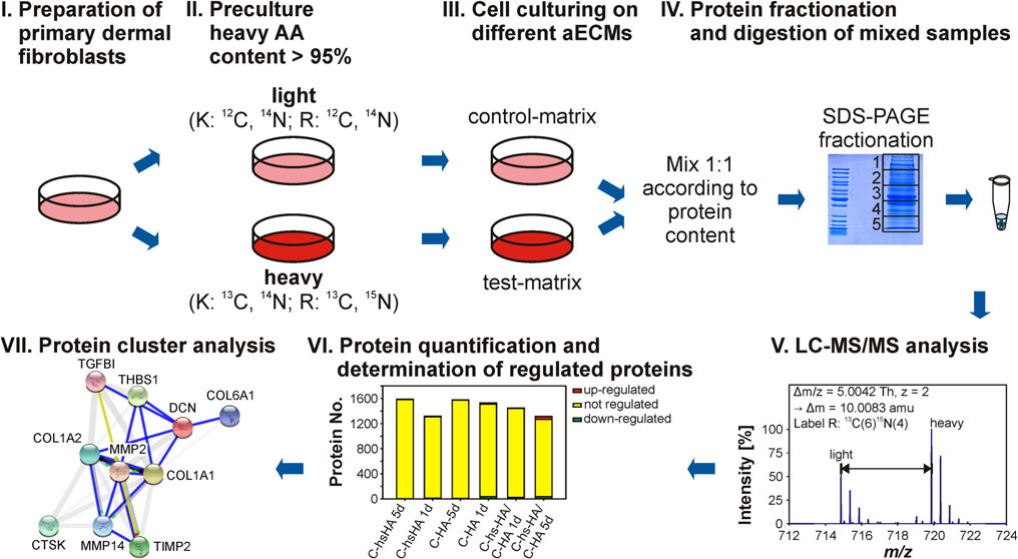
Experimental workflow. **I** Primary dermal fibroblasts are prepared from healthy female donors. **II** Primary dermal fibroblasts are precultured either in light medium (L) or heavy medium (H) containing isotopically labeled lysine and arginine until heavy amino acid content is larger than 95 % in the proteins. **III** Cells are cultured on different aECMs. Control-matrix and test-matrix have different isotope labeling. **IV** After culturing for 1 respectively 5 days, cells are harvested, lysed and mixed 1:1 according to their protein content. Proteins are fractionated by SDS-PAGE and digested in-gel by trypsin. **V** Peptides are analyzed by LC–MS/MS. **VI** MS data is processed by Maxquant. Pairs of light and heavy labeled peptides enable relative protein quantification. Regulated proteins are determined. **VII** Proteins considered to be regulated are subjected to bioinformatics tools like DAVID and PANTHER for cluster analysis according to biological processes and molecular function (cluster diagram was made on http://string-db.org/)

An overview of the experimental workflow after isolation of primary dFb is shown in Fig. 1. For isotope labeling dFb were cultivated in SILAC DMEM (Pierce SILAC Protein Quantitation Kit—DMEM, Pierce Biotechnology, Rockford, USA) containing either 0.798 mmol/l heavy ^13^C^14^N lysine and 0.398 mmol/l heavy ^13^C^15^N arginine (heavy medium) or ^12^C^14^N lysine and ^12^C^14^N arginine (light medium) supplemented with 10 % dialyzed FCS for 10 days on polystyrene (PS) culture plates with medium change every 2 days.

4.0 × 10^5^ (24 h exposure) and accordingly 1.5 × 10^5^ cells (5 days exposure) were transferred to 75 cm^2^ cell culture flasks coated with different aECMs consisting of rat tail collagen type I (C) (BD Bioscience, Heidelberg, Germany) and HA (Aqua Biochem, Dessau, Germany) or hsHA (provided by Innovent e.V., Jena, Germany) described by van der Smissen et al. [20] and incubated for 1 or 5 days. At the time point 5 days the monolayer appeared with a donor dependent confluence of 70–100 %. One day incubation was meant to determine immediate cell responses to hsHA, whereas 5 days of incubation should reflect changes in the proteome of almost confluent grown dFbs. The appropriate culture variations are listed in Table 1. These variations offer the comparison of light and heavy labeled cells after the cultivation on the different aECMs.

**Table 1:** aECMs, according abbreviations and the applied culture medium and incubation time

aECM (abbreviation)	SILAC labeling medium	Incubation time (days)
PS control matrix replicates 1 + 2	Light medium	1
		5
	Heavy medium	1
		5
Collagen type I (C) control matrix replicates 3 + 4	Light medium	1
		5
	Heavy medium	1
		5
Collagen type I/hyaluronan (C-HA)	Light medium	1
		5
Collagen type I/C-hsHA	Heavy medium	1
		5

Proteomic analysis was carried out on the basis of cell lysates. Therefore, fibroblasts were harvested after days 1 or 5 post seeding by addition of 0.25 % EDTA (Sigma, St. Louis, MO, USA) in 1× PBS (PAA) to prevent damage of integrins by trypsin. The cell pellet was stored on ice and washed three times with cold 1× PBS before the final centrifugation for 6 min, 12,000 rpm at 4 °C. The supernatant was discarded and cell pellet immediately frozen at −80 °C until further use.

Harvested cells were disrupted in 100 μl lysis buffer containing 6 M urea, 2 M thiourea and 100 mM ammonium bicarbonate by vortexing for 3 min. Cell debris and undissolved material were removed by centrifugation (16,000×*g*, 10 min, 18 °C). Protein concentration of the supernatants was measured using Quick Start Bradford Protein Assay (Biorad, Hercules, CA) with bovine serum albumin as reference. Samples gained from the different aECMs were combined at 1:1 (w/w) protein ratio with the appropriate control (PS or C).

### SDS-PAGE and in-gel digestion of proteins

In order to increase the amount of quantified proteins, samples were fractionated using sodium dodecyl sulfate polyacrylamide gel electrophoresis (SDS-PAGE). For the gel separation, 15 μg protein of each sample were mixed 3:1 with 4× Laemmli sample buffer (12 % [w/v] SDS, 6 % [v/v] β-mercaptoethanol, 30 % [w/v] glycerol, 150 mM Tris–HCl [pH 7.0], 0.04 % [w/v] bromphenol blue) and incubated 1 h at 37 °C. Protein separation was performed by 12 % SDS-PAGE with a 4 % stacking gel. Gel electrophoresis was stopped after proteins entered approximately 3 cm in the gel. The Coomassie staining procedure was performed according to Müller et al. [27].

The protein lanes were cut in five equal gel slices. In-gel digestion of protein was performed similar to Mörbt et al. [28] with 100 ng trypsin per slice (trypsin sequencing grade from bovine pancreas, Roche, Mannheim, Germany). Samples were concentrated by vacuum centrifugation and reconstituted with 0.1 % (v/v) formic acid after tryptic digestion.

### Liquid chromatography tandem MS analysis

Tryptic peptides from in-gel digestion were separated by nano-high performance liquid chromatography (nano-HPLC) prior to mass spectrometry (MS) analysis to increase the number of quantified peptides and corresponding proteins. Liquid chromatography tandem MS analysis was performed according to Müller et al. [27] with some slight modifications. Peptides were analyzed with a nano-HPLC system (nanoAquity, Waters, Milford, MA, USA) coupled online with an LTQ Orbitrap XL mass spectrometer (Thermo Fisher Scientific, San Jose, CA, USA) via a nano-electrospray ion source (TriVersa NanoMate, Advion, Ithaca, NY, USA). Samples were injected on a trapping column (nanoAquity UPLC column, C18, 180 μm × 20 mm, 5 μm, Waters) and washed with 2 % acetonitrile containing 0.1 % formic acid and a flow rate of 15 μl/min for 8 min. A C18 UPLC column (nanoAcquity UPLC column, C18, 75 μm × 150 mm, 1.7 μm, Waters) was used for peptide separation. Peptides were eluted using a gradient from 2 to 85 % acetonitrile, 0.1 % formic acid (0 min, 2 %; 2 min, 2 %; 7 min, 6 %; 55 min, 20 %; 73 min, 30 %; 91 min, 40 %; 94 min, 85 %) with a flow rate of 300 nl/min and a column temperature of 40 °C.

MS analysis was performed with a spray voltage of 1.8 kV in positive ion mode. The mass spectrometer automatically switched between full scan MS mode (from 400 to 1,400 *m*/*z*, *R* = 60,000) and MS^2^ acquisition. Peptide ions exceeding an intensity of 5,000 counts were fragmented within the linear ion trap by collision induced dissociation (isolation width 4 *m*/*z*, normalized collision energy 35, activation time 30 ms, activation Q 0.25). A dynamic precursor exclusion of 3 min for tandem MS measurements was applied.

### Data analysis

Protein identification and relative quantification was carried out with the software MaxQuant [29] (version 1.2.0.18, Max Planck Institute of Biochemistry, Munich, Germany). Peptides with the same sequence but different labeling states elute at the same retention time. Heavy to light peptide pairs can be detected by their distinct mass shifts according to the labeling with heavy arginine and lysine. MaxQuant uses the intensity of heavy and light labeled peptide pairs to calculate relative peptide abundances. The derived peptide intensity ratios belonging to the same protein are the basis for relative protein quantification.

Within the MaxQuant workflow, database searching was carried out by the Andromeda search engine [30] against a reverse concatenated IPI human database (version 3.68) including a contaminant list. Recalibration of precursor masses by the option “first search” with a 20 ppm mass tolerance against the human first search database provided by MaxQuant.org. Trypsin with maximum two missed cleavages was set as protease. Carbamidomethylation of cysteine was specified as fixed modification, and oxidation of methionine and acetylation of the protein N-terminal were defined as variable modifications. A peptide mass tolerance of 6 ppm was applied. For tandem MS identification six top peaks per 100 Da were chosen and searched with a fragment ion mass tolerance of 0.5 Da.

Peptide and protein false discovery rates were limited to 1 %. Protein identification required at least two unique peptides. The minimal peptide length was set to six amino acids. For protein quantification, the minimal peptide ratio count was set to 2. The option “match between runs” was used for samples measured within the same batch. Re-quantification of proteins was also applied.

Proteins with a log_2_ fold change (FC) above 0.5 or below −0.5 were considered to be up- respectively down-regulated. Furthermore, only proteins showing in at least three out of four replicates regulation in the same direction and an average FC of all replicates fulfilling the criteria for regulation were considered as significantly regulated.

For identification of significantly regulated clusters of functionally related regulated proteins the web-based bioinformatics tool DAVID [24] was used. The list of regulated proteins was subjected to DAVID, whereas all identified proteins served as background for cluster analysis. Protein clustering was performed according to biological and molecular function derived from the PANTHER classification system [24].

### Control experiments for significance estimation of regulation thresholds

Experiments with primary cells often show large variation between different donors. Additionally, technical variance is another error source. With regard to these issues, we tested the significance of our regulation thresholds with two control experiments. Each control experiment was performed in triplicates. The first experiment was to evaluate the labeling effect of the SILAC experiments. Therefore, protein samples of the same donor from cells grown on light and heavy medium with collagen type I as matrix were mixed 1:1 (w/w) according to their protein content. The second control experiment examined the donor effect and included protein samples from three donors. Therefore, heavy and light labeled protein samples of different donors were mixed 1:1 (w/w) according to their protein content (donor A heavy + donor B light, donor B heavy + donor C light, donor A light + donor B heavy). Further treatment and measurement was similar to the other samples. Proteins with a log_2_ FC larger than 0.5 or lower than −0.5 were defined as regulated.

### Western blot and zymography

Data analysis with MaxQuant and the bioinformatics tool DAVID resulted in a set of regulated protein clusters. Selected proteins belonging to regulated clusters were chosen for further confirmation by western blotting or zymography. Western blots of cell lysates were performed with antibodies against MMP-14, TSP-1, collagen types I and VI (α chain 1). The enzymatic activity of MMP-2 in the culture supernatant was tested by gelatine zymography [26] to investigate whether altered MMP-2 expression leads to activity changes.

3.5 × 10^5^ cells were seeded on aECM provided in petri dishes (94 mm diameter) and incubated for 72 h with DMEM/10 % FCS, another 24 h with DMEM/0 % FCS to generate serum free supernatants and additional 24 h with DMEM/10 % FCS to gain an incubation time of 5 days in total. Samples from six different donors were applied for validation by western blotting and zymography.

Cell extracts were prepared by detaching cells with 0.05 % trypsin/0.02 % EDTA (Biochrom, Berlin, Germany) and cooled lysis of cell pellets with RIPA-buffer (50 mM HEPES, 150 mM NaCl, 5 mM EDTA, 1 mM EGTA, 1 % Triton X-100, 0.1 % SDS, 1 % deoxycholate, 1 mM dithiothreitol [Roth, Karlsruhe, Germany; Serva, Heidelberg, Germany; Sigma, Taufkirchen, Germany]). Protein lysates were separated by SDS-PAGE with appropriate SDS gels (Amersham ECL gels, GE Healthcare, München, Germany) and blotted on OPTITRAN BAS83 membrane. Primary antibodies for MMP-14 (rabbit-anti-human, clone ID: EP1264Y, Epitomics, Burlingame, USA), TSP-1 (rabbit-anti-human, Abcam, Cambridge, United Kingdom), collagen type VI α chain 1 (rabbit-anti-human, Atlas Antibodies, Stockholm, Sweden), collagen type I α 1 (rabbit-anti-human, Sigma) and GAPDH (mouse-anti-human, Merck-Millipore, Darmstadt, Germany) were combined with IRDye 680RD goat-anti-rabbit or IRDye 680RD goat-anti-rabbit (LI-COR, Lincoln, USA) as secondary antibodies.

Cell-free supernatants were concentrated by ultrafiltration using vivaspin six columns (GE Healthcare) for MMP-2 gelatine zymography [26]. An amount of 5 μg of concentrated supernatant was diluted in a sample buffer (0.3 M Tris–HCl pH 8.8, 4 % saccharose, 10 % SDS and 0.1 % bromphenol blue), applied to a 10 % SDS-gel containing, 0.1 % gelatine, and was electrophoretically separated. After electrophoresis, gels were washed in 2.5 % Triton X-100 for 30 min and were incubated overnight at room temperature in a development buffer containing 0.05 M Tris–HCl, pH 8.0, 8 mM CaCl_2_. MMP-2 associated gelatine digestion was visualized as white bands in the gel after staining with 0.1 % Coomassie blue R250 and clearing with 7.5 % acetic acid. MMP-2 activity was quantified by densitometric measuring (Intas, Göttingen, Germany). The absolute integrated area under the peak was determined.

## Results

### Significance estimation of regulation thresholds

In order to estimate the effects of technical variance during cell culture (labeling effect) and the biological variance caused by different donors (donor effect), we set up two control experiments. Samples from three different donors were used for the significance estimation.

To investigate the labeling effect, cells from three different donors were split up and cultivated in either heavy (containing ^13^C^14^N lysine and ^13^C^15^N arginine) or light SILAC medium. Heavy and light stable isotope labeled cells of the same donor were lysed, mixed and analyzed. Analogously the donor effect was determined by mixing differentially labeled samples of the different donors. Between 600 and 900 proteins were quantified by MaxQuant. Analysis of labeling effect resulted in average 0.6 % of all identified proteins fulfilling the up-regulation threshold, whereas 5.9 % pass the threshold for down-regulation. This is clearly showing that the abundance of light labeled proteins is overestimated during protein quantification process even so typical contaminants such as keratins, trypsin as well as rat collagen type I, which was used as aECM component, were defined as contaminants and thus discarded during the quantification process.

Six proteins are found to be regulated in all three replicates with a log_2_ FC less than −0.5. Namely, two Ras-related proteins (RAB2, RAB5), histone H1.2, dermcidin and collagen type I α chains 1 and 2 are fulfilling the threshold in all samples. The fact that all of these proteins are showing a higher abundance of light labeled protein in this control experiment indicates that these proteins can be classified as contaminants. Dermcidin for example is a 91 amino acid long antimicrobial peptide secreted by perspiratory glands which can occur as a contaminant. Even rat collagen type I was already inserted to the contaminant list of MaxQuant, the abundance of light labeled human collagen type I is higher than the heavy labeled counterpart in this control experiment. Only unique peptides were accepted for calculation of heavy to light ratios. Which means that collagen type I contamination has to stem from another source than the applied aECM.

The donor effect was estimated by measuring a mixture of heavy and light control samples of different donors. On average 12.9 % of all identified proteins show a log_2_ FC less than −0.5, and 8.2 % have a FC larger than 0.5. To evaluate whether this donor effect is random or not, only proteins which had the same direction of FC in all replicates were used for further analysis. Only 0.1 % of proteins identified in all replicates fulfilled the threshold criteria and have the same direction of FC. This demonstrates that variability of protein abundance by different donors is exclusively a random effect.

To estimate the false positive rate (FPR) of regulated proteins in our SILAC experiment, we used a stochastic equation based on combinatorics. The FPR is calculated by summing up, that three out of four or four out of four measurements are representing a regulation by chance in the same direction. As probability for false positive up- or down-regulation we took the experimental values derived from the donor effect measurements as it showed the largest variability (*p* = 8.2 %, *q* = 12.9 %).

1 $$ {\text{FPR}} = \left( {\begin{array}{*{20}c} 4 \\ 3 \\ \end{array} } \right) \cdot p^{3} \cdot (1 - p) + \left( {\begin{array}{*{20}c} 4 \\ 4 \\ \end{array} } \right) \cdot p^{4} + \left( {\begin{array}{*{20}c} 4 \\ 3 \\ \end{array} } \right) \cdot q^{3} \cdot (1 - q) + \left( {\begin{array}{*{20}c} 4 \\ 4 \\ \end{array} } \right) \cdot q^{4} < 1\,\% $$

With Eq. 1 a FPR lower than 1 % was calculated for the chosen protein regulation thresholds demonstrating high significance.

### Classification of quantified proteins

**Fig. 2:**
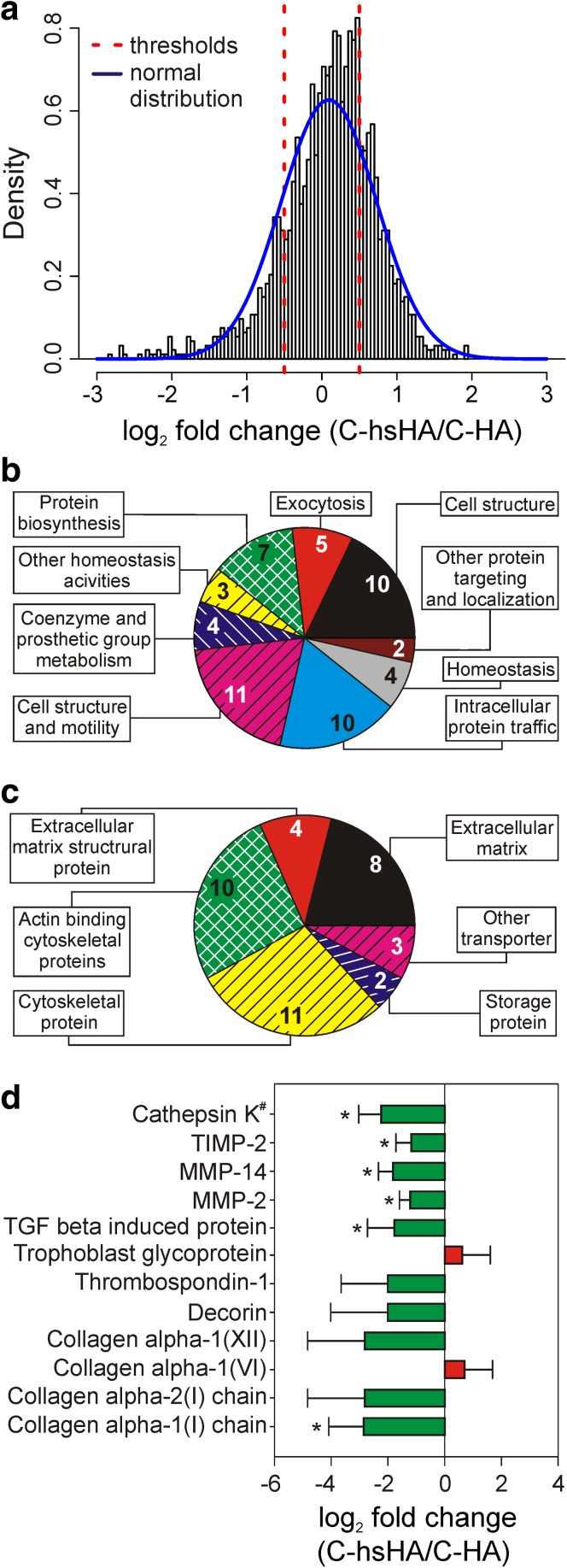
Cluster analysis of proteins regulated by HA sulfation at day 5 post seeding. **a** The log_2_ FC between the matrices C-hsHA and C-HA is plotted against the density. FCs show a normal distribution around zero. **b** Clustering of proteins regulated by HA sulfation according to PANTHER biological processes. **c** Clustering of proteins regulated by HA sulfation according to PANTHER molecular functions. **d** FCs of proteins clustered by DAVID according to PANTHER biological processes and molecular function (*MF00178* ECM, *MF00179* ECM structural protein, *BP00124* Cell adhesion). *T test *P* value <0.05. ^#^catK was added manually to the cluster according to its collagen degrading function in the lysosomes

In the main proteomic experiments cellular response to the different aECMs (C-HA and C-hsHA) after different incubation times (1 or 5 days) was investigated. Overall 2,419 proteins were quantified. Between 61 and 70 % of these proteins were quantified in at least three out of four biological replicates. Cell compartment classification of the identified proteins was done according to gene ontology (GO) annotations using the software STRAP (Software Tool for Rapid Annotation of Proteins) [31] (Supplementary Table 1). Most proteins were assigned to the cytoplasm (35 %) followed by the nucleus (34 %) and the plasma membrane (15 %). Since we found only a few regulated proteins for C-HA (1.9 %) but a more prominent effect for C-hsHA (3.6 %), we calculated the ratio between C-hsHA and C-HA at corresponding time points (Table 2). Thus we eliminated the effect of the control matrix by dividing the ratios of C-hsHA and C-HA. This is supported by the normal distribution around zero in the density plot of log_2_ FCs for C-hsHA related to C-HA at day 5 post seeding (Fig. 2a). The fraction of regulated protein was between 2 (C-hsHA/C-HA day 1) and 6.7 % (C-hsHA/C-HA 5 days) (Table 2).

**Table 2:** Protein quantifications on C-HA and C-hsHA at 1 and 5 days post seeding

	C-HA 1 days	C-HA 5 days	C-hsHA 1 days	C-hsHA 5 days	C-hsHA/C-HA 1 days^a^	C-hsHA/C-HA 5 days^a^
Total protein quantifications	2262	2150	2244	2224	2109	1885
Proteins quantified in ≥3 replicates	1589 70 %	1318 61 %	1575 70 %	1529 69 %	1448 69 %	1213 64 %
Down-regulated	0	13	7	36	24	38
Up-regulated	7	12	1	18	9	46
Regulated proteins (%)	0.44	1.90	0.51	3.53	2.28	6.92

^a^Values for C-hsHA/C-HA are calculated by measured ratios of C-HA and C-hsHA at corresponding time points

Proteins fulfilling the regulation thresholds were clustered using the web-based tool DAVID [25] according to their molecular functions respectively their biological process using the PANTHER GO database [24]. The cluster analysis revealed one significant cluster (enrichment score >1.5) for the comparison of C-hsHA and C-HA at day 5 post seeding. Ten regulated proteins were associated to the ECM and cell adhesion (MF00178, MF00179, BP00124). Based on the low number of regulated proteins on day 1 post seeding, no significant clusters could be determined. Classification and clustering of regulated proteins according to the PANTHER database is shown in Fig. 2.

### Effects of HA sulfation on the expression of ECM and cell adhesion related proteins

Bioinformatics analysis with DAVID shows regulation of 10 proteins associated with ECM (PANTHER cluster: MF00178, MF00179, BP00124) at day 5 (Fig. 2) according to HA sulfation. We manually added catK to the ECM cluster since it is an important protein for collagen degradation [32].

**Fig. 3:**
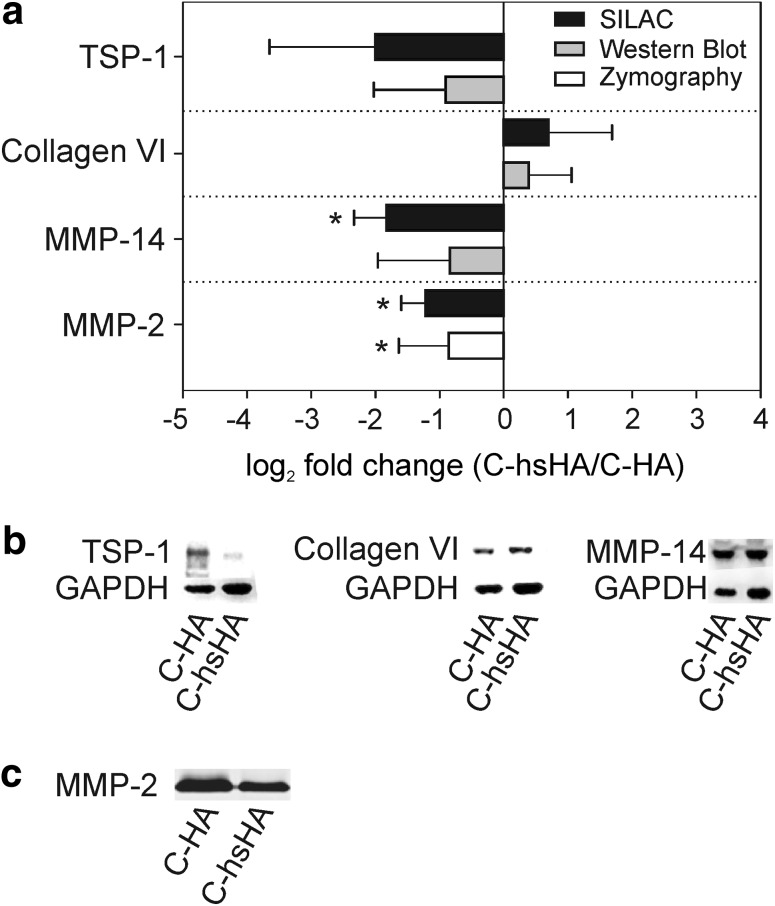
Validation of selected proteins regulated by HA sulfation at day 5 post seeding by western blotting and zymography. **a** Comparison of log_2_ FC values derived by SILAC, western blotting and zymography. *T test *P* value <0.05. **b** Representative western blots. **c** Representative MMP-2 zymography of culture supernatant

The regulated proteins MMP-14, collagen types I, VI and TSP-1 were chosen to confirm the SILAC results by western blotting (Fig. 3; Supplementary Table 2). MMPs-2, -14, collagen type VI and TSP-1 showed the same regulation as revealed by SILAC analysis. On the other hand, collagen type I western blots could not verify down-regulation of collagen type I on C-hsHA after 5 days of exposure.

Additionally, MMP-2 zymography was performed to measure the relative activity in the culture supernatants. Both, protein expression determined by SILAC and MMP-2 activity in the culture supernatant are diminished by HA sulfation.

## Discussion

Previous investigations indicated that matrices with sulfated GAGs modulate cellular responses like cell adhesion, cell proliferation or matrix production [20]. In this study, we set up a SILAC experiment to extend knowledge about protein regulation caused by sulfation of HA with an untargeted approach. We focused on fibroblasts since these cells are crucial for wound closure and synthesis of new tissue.

We used primary dFb from healthy individuals in our experiments to examine effects on the proteome as close as possible to the in vivo situation. This is indispensable if the results should be referred to the original cell metabolism. For example Pan et al. [33] showed that a hepatoma cell line had up-regulated cell-cycle associated functions and down-regulation of drug metabolism compared to their cognate primary cells. Contrary to our results, Abatangelo et al. [34] reported that soluble hsHA (substitution degree 3) had no growth promoting effect on a mouse fibroblast cell line (NTC L929). This result might also be caused by the usage of an immortalized cell line. However, a clear drawback of experiments with primary cells lies in their higher biological variance compared to cell lines.

SILAC is a well-established method to relatively quantify the abundance of proteins in a shotgun approach. It is well suited for experiments with primary cells because control and treated sample from the same donor are compared within one measurement. Therefore, the effect of the donor is minimized. Nevertheless, experiments with primary cells cause high variance of results. In order to cope with this, we applied an extended set of controls for the labeling as well as the donor effect. The results are showing that for primary dFb, SILAC can be used to investigate changes in the proteome with a FPR lower than 1 % with the applied criteria.

**Fig. 4:**
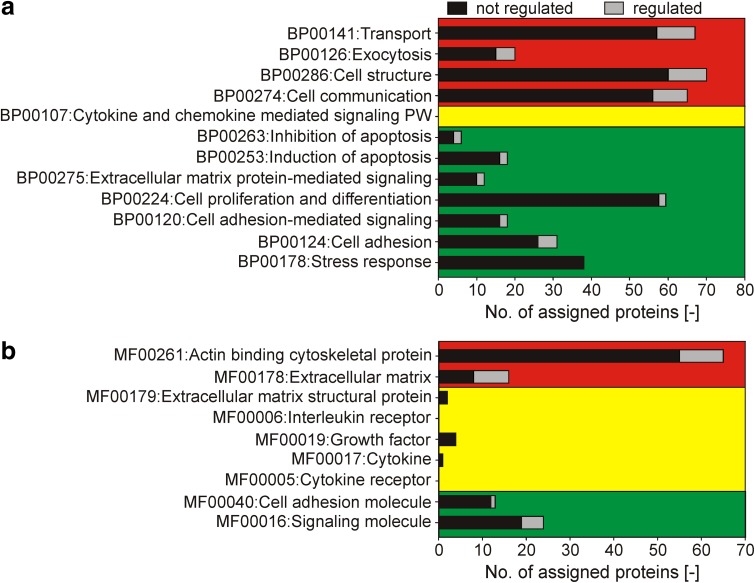
Comparison of regulated and non-regulated relevant protein clusters by HA sulfation according to PANTHER **a** biological processes and **b** molecular function at day 5 post seeding. The *bars* indicate the number of identified proteins which were regulated (*gray*) or not regulated (*black*) for each protein cluster. The two *graphs* are divided in three *boxes*. Regulated protein clusters are in the *upper boxes* (*red*). Protein clusters with less than five proteins are in the *middle boxes* (*yellow*). Protein clusters which are not regulated and include more than five protein identifications are in the *lower boxes* (*green*) (Color figure online)

As expected the applied aECMs showed good biocompatibility which is in line with toxicity studies for sulfated HA [34]. Our previous results already showed that sulfation of HA increases cell adhesion and proliferation [20]. The good biocompatibility is reflected by the fact that there were no significantly regulated clusters detectable after 24 h of culture. In order to allow future work to focus on the really relevant pathways and molecular functions in terms of effects of aECM, we summarized the results of gene enrichment analyses in Fig. 4. The figure shows relevant protein clusters with information about significant enrichment of regulated proteins. The diagram highlights that neither apoptosis nor stress response are regulated by HA sulfation. Thus any cell activation or danger programs are excluded for the application of C-hsHA. However, proteins in those relevant clusters could be selected and used for fast and reliable detection with targeted approaches like selected reaction monitoring [35], western blotting or enzyme linked immunosorbent assay [36].

**Fig. 5:**
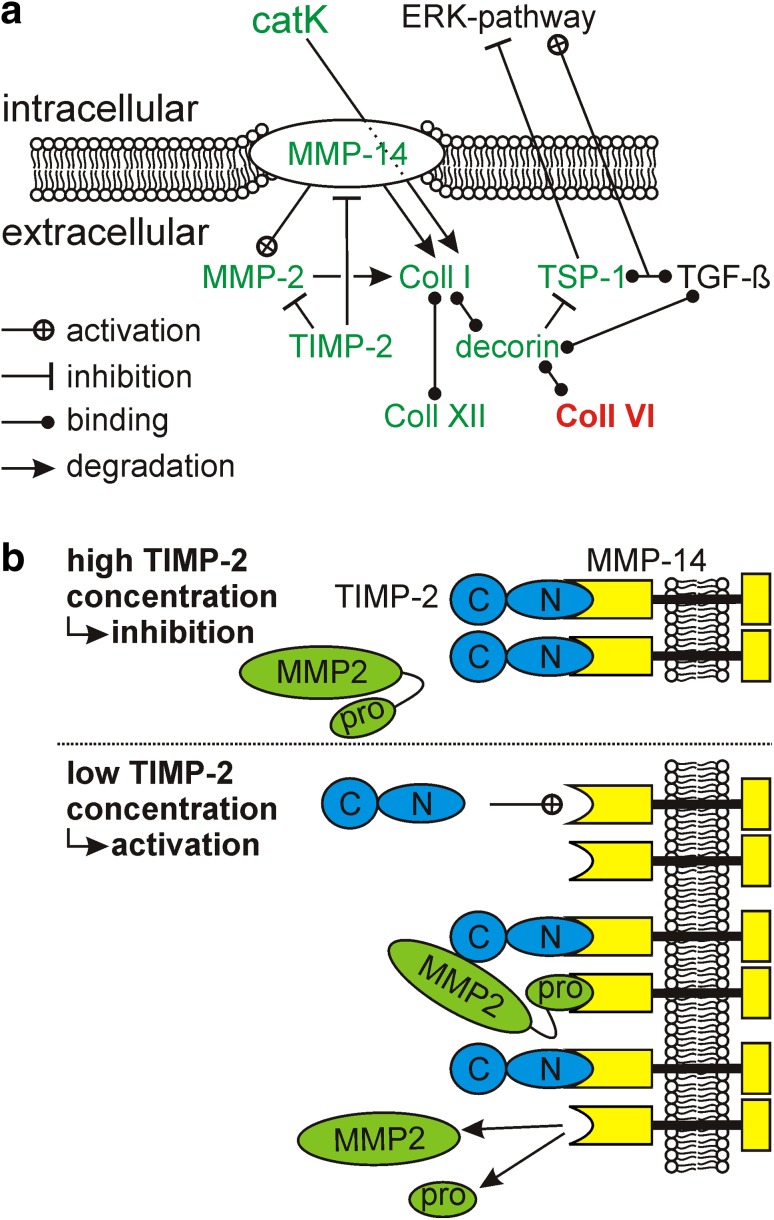
Biological processes and the relationship between proteins in the regulated ECM associated cluster. **a** Scheme of protein relationships in a biochemical context. The connection *lines* between the different proteins indicate activation, inhibition, binding, or degradation of associated proteins or pathways. Proteins, which were found to be down-regulated on C-hsHA are *green*, whereas up-regulated proteins are marked *bold red*. **b** Influence of TIMP-2 on activation of MMP-2 according to the proposed mechanism by Nagase et al. [61]. High concentrations of TIMP-2 inhibit proMMP-2 conversion by blocking the active site of MMP-14. On the other hand, low concentrations of TIMP-2 are required for MMP-2 activation. TIMP-2 binds to MMP-14 with its N-terminal domain. In a second step proMMP-2 is recruited by MMP-14 bound TIMP-2. Closely located free MMP-14 binds proMMP-2 and cleaves the propeptide to activate MMP-2 (Color figure online)

We focused on the significant clusters to show relevant effects caused by HA sulfation. The gene enrichment analyzes resulted in a clear enrichment in terms of cell adhesion and regulation of the ECM. The biochemical relationship between members of this cluster are shown in Fig. 5a.

Collagen type I, which is the main type in the dermal ECM [37], is the first member of the regulated protein cluster (Fig. 2). Collagen type I gives tensile strength to skin and bone tissue [38]. It replaces destroyed dermal tissue and is deposited mainly by myofibroblasts upon stimulation by TGF-β [39]. Western blotting could not confirm decreased collagen type I expression in this study. Nevertheless, the previous study of van der Smissen et al. [20] support the results derived by SILAC.

Collagen type XII protein expression is also reduced in response to hsHA. It is localized at the surface of collagen fibrils and acts as a bridge between them [40]. Increased expression of collagen type XII by dFbs is known to promote collagen type I gel contraction [41]. Thereby deformability is decreased and migration of dFb into the ECM is inhibited [40]. dFbs produce more collagen type XII when they grow on attached compared to floating collagen type I gels [42], but the underlying mechanism is not discovered by now.

On the other hand, cells on C-hsHA express higher levels of collagen type VI. This ECM compound is known to be produced by dFb when they get confluent to generate an appropriate cell environment [43].

TSP-1 is also down-regulated for C-hsHA. The expression of TSP-1 is increased in response to tissue damage, inflammation, or growth factors like platelet derived growth factor, TGF-β and basic FGF [44, 45]. Freshly synthetized TSP-1 gets integrated in the ECM or binds to the cell surface, where it is quickly internalized and degraded [46]. TSP-1 has the ability to activate TGF-β and to inhibit angiogenesis [45, 47, 48]. It is also known to influence adhesion, migration, cytoskeletal organization and apoptosis of cells by interaction with different cell receptors [45]. Thereby the mode of TSP-1 action strongly depends on the cell type and its cell surface receptors. For example smooth muscle cell migration is induced [45], while essential signal cascades like the extracellular signal-regulated kinase (ERK) pathway are inhibited by TSP-1 [49]. The ERK pathway includes a phosphorylation cascade of different proteins in response to growth factors, cytokines or hormones. It controls different cell functions like cell proliferation, differentiation and apoptosis [50]. Aberrant activation of the ERK pathway is present in many cancers [51].

In our study, TSP-1 abundance was lower for the C-hsHA matrix which had pro-proliferative properties on dFb in a previous study [20]. Hence, TSP-1 can promote proliferation when TGF-β is bound whereas unbound TSP-1 reduces proliferation by inhibition of the ERK pathway (Fig. 5a). C-hsHA strongly binds TGF-β [52] and thereby prevents TGF-β-signaling in fibroblasts grown on C-hsHA (Anderegg U, personal communication). Therefore, TSP-1 might be less effective on C-hsHA in addition to its decreased expression observed here.

Additionally, decorin was found to be down-regulated for C-hsHA. This proteoglycan with attached chondroitin and dermatan sulfate chains interacts with many proteins of the regulated ECM cluster. Two different binding sites related to collagen fibrils enable decorin to bridge collagen types I and VI [53] (Fig. 5a). Decorin has also the ability to bind to collagen type XII [54]. It is essential for ECM cross-linking since decorin deficient mice produce abnormally fused collagen bundles which lead to increased skin fragility [55]. On the other hand, cell attachment to TSP-1 is inhibited by decorin through binding to its cell adhesive site [56] (Fig. 5a). Decorin is also important for binding different growth factors like FGF-2 with its sulfated GAG chains [57].

The MMPs-2 and -14 (also named MT1-MMP) and their inhibitor tissue inhibitor of metalloproteinases 2 (TIMP-2) build a complex regulation network for collagen degradation during wound healing. Besides collagen type I, membrane bound MMP-14 has a huge variety of different substrates including laminin, lumican, integrin αV, transglutaminase, CD44H, syndecan 1 and IL-8 [58]. Collagen fibers are degraded by MMP-14 in short fragments which are further degraded intracellular by phagocytosis involving catK [32, 59].

MMP-2 is secreted in its inactive form proMMP-2 and gets activated by MMP-14 (Fig. 5b) [60, 61]. Lee et al. [59] showed that MMP-14 but not MMP-2 is necessary for phagocytosis of collagen type I. Indeed, MMP-2 is able to cleave interstitial but not helical collagen type I [62]. Thus MMP-14 is the key enzyme for collagen phagocytosis. TIMP-2 is an inhibitor of both MMPs-2 and -14. Interestingly, activation of proMMP-2 by MMP-14 is enhanced by a low amount of TIMP-2, whereas higher concentrations lead to inhibition of MMP-14 [63] (Fig. 5b). Additionally, blocking of TIMP-2 by an antibody abrogates MMP-2 activation [63, 64]. Moreover, HA has also the ability to induce proMMP-2 activation [65]. Sulfated HA might not have the ability to induce proMMP-2 activation, which results in lower abundance of active MMP-2 for cells grown on C-hsHA.

CatK is also related to ECM degradation processes due to its ability to degrade collagens, elastins and proteoglycans [66]. Collagens are degraded after endocytosis in the lysosomes where catK is highly expressed [32]. CatK is usually not expressed in healthy skin, while its expression is induced by inflammation or in scar formation [32, 66]. For example it is up-regulated in synovial fibroblasts, which are key players in rheumatic arthritis because of their cartilage degrading activity [66]. In our experiment MMPs-2, -14, TIMP-2 and catK are down-regulated when comparing the aECMs C-hsHA and C-HA. Furthermore previous results showed, that MMP-1 is significantly down-regulated on mRNA level for C-hsHA [20] suggesting altogether that matrix remodeling is diminished by hsHA. This hypothesis is strengthened by the down-regulation of collagen types I and XII expression. Cells growing on non-sulfated matrix might degrade the provided aECM and build up their own matrix according to their requirements.

Interestingly, therapeutic wound dressings which result in an reduced ECM degradation or direct inactivation of MMPs are known to improve healing of chronic skin wounds since disorders in the MMP–TIMP balance can lead to fibrosis, metastasis or tumor growth [37]. There are several clinical products on the market, which target MMPs to rebalance the wound environment and to improve healing of chronic wounds. Promogran^®^ for example consists of oxidized regenerated cellulose and collagen which binds and inactivates MMPs [67]. The product Fibracol^®^ also reduces the activity of MMPs by competitive inhibition with collagen [68]. A formulation of metal ions and citric acid is used in DerMax^®^ wound dressings to reduce oxygen free radicals and MMP-2 activity [69, 70].

In conclusion, introduction of sulfate groups in HA of growth substrates influences the expression of MMPs and other ECM related proteins which are involved in ECM remodeling by dFbs. These effects occur without induction of stress, promising good biocompatibility of hsHA. Especially, considering the described positive effects on healing of chronic wounds by inhibition of MMPs along with increased proliferation [20] and the low cellular stress level further encourages the application of hsHA as an appropriate therapeutic agent in wound dressings.

Our study shows that quantitative proteomics is a valuable tool for unbiased evaluation of aECM effects. It can be used to preselect suited aECM prior to animal testing. Moreover, the untargeted protein analysis provides a set of biological markers and pathways for further detailed investigations. Thereby animal experiments can be reduced to promising aECMs for clinical application. Nevertheless, in vitro experiments cannot completely simulate the situation in vivo. Ultimately further investigations of aECMs in animal experiments are indispensable to proof their influence on wound healing and long term effects.

## Electronic supplementary material

Below is the link to the electronic supplementary material.

Supplementary material 1 (PDF 49 kb)

Supplementary material 2 (XLSX 1558 kb)

Supplementary material 3 (XLSX 3686 kb)
