# Supplementary material for: Quantitative proteomics reveals altered expression of extracellular matrix related proteins of human primary dermal fibroblasts in response to sulfated hyaluronan and collagen applied as artificial extracellular matrix
Source: J Mater Sci Mater Med. 2012 Sep 19;23(12):3053–65. doi: 10.1007/s10856-012-4760-x (PMC3506194; doi:10.1007/s10856-012-4760-x)
Supplement: Supplementary file 1 — Supplementary material 1 (PDF 49 kb) [file 10856_2012_4760_MOESM1_ESM.pdf]

Quantitative proteomics reveal altered extracellular matrix remodeling of human primary dermal fibroblasts in response to sulfated hyaluronan and collagen applied as artificial extracellular matrix

Stephan A. Müller[a,d], Anja van der Smissen[b,d], Margarete von Feilitzsch[b,d], Ulf Anderegg[b,d], Stefan Kalkhof[a,d] and Martin von Bergen[a,c,d]

[a] *Department of Proteomics, UFZ, Helmholtz-Centre for Environmental Research Leipzig, 04318 Leipzig, Germany*

[b] *Department of Dermatology, Venerology and Allergology, Leipzig University, 04103 Leipzig, Germany*

[c] *Department of Metabolomics, UFZ, Helmholtz-Centre for Environmental Research Leipzig, 04318 Leipzig, Germany*

[d] *Collaborative Research Center (SFB-TR67), Matrixengineering Leipzig and Dresden, Germany*

Corresponding Author

Martin von Bergen

Phone: +49-341-235-1211

Fax: +49-341-235-451211

Email: Martin.vonbergen@ufz.de

Supplementary table 1: Protein cell compartment classification for identified proteins according to GO annotations using STRAP [1]

Cytoplasm	Macromolecular Complex	Other intracellular organelles	Extracellular	Cell Surface	Plasma Membrane	Cytoskeleton	Peroxisome/microbody	Nucleus	Mitochondria	Endoplasmatic Reticulum	Ribosome	Chromosome	Endosome	Other
857	201	313	155	31	359	271	19	825	301	216	122	75	108	466
35%	8%	13%	6%	1%	15%	11%	1%	34%	12%	9%	5%	3%	4%	19%

Supplementary table 2: Regulated proteins between C-hsHA and C-HA related to extracellular matrix and cell adhesion on day 5. * indicates proteins with a p-value < 0.05. # indicates proteins that were not clustered by DAVID although they are strongly related to ECM degradation

Protein Name	Uniprot accession	Log_2 Fold Change	Standard deviation	T-test p-value
Matrix metalloproteinase 2 (MMP-2)*	P08253	-1.22	0.38	0.03
Matrix metalloproteinase 14 (MMP-14)*	B2R6P3	-1.82	0.51	0.02
Decorin	P07585	-2.01	2.01	0.14
Thrombospondin-1 (TSP-1)	P07996	-2.00	1.64	0.09
Collagen α-1(I) chain*	P02452	-2.86	1.23	0.02
Collagen α-2(I) chain	P08123	-2.81	2.02	0.07
Collagen alpha-1(VI)	P12109	0.70	0.99	0.25
Collagen alpha-1(XII)	P08123	-2.81	2.02	0.07
Trophoblast glycoprotein	Q13641	0.62	0.98	0.29
Metalloproteinase Inhibitor 2 (TIMP2) *	P16035	-1.18	0.54	0.02
Transforming growth factor beta induced protein	Q15582	-1.77	0.95	0.03
Cathepsin K (CatK)*#	P43235	-2.24	0.79	0.04

Supplementary table 3: Validation of selected proteins by western blotting ([1]) or zymography ([2]) related to extracellular matrix and cell adhesion after 5 day exposure on C-hsHA.

Protein Name	Uniprot accession	Log_2 Fold Change	Standard deviation	T-test p-value
Matrix metalloproteinase 2 (MMP-2)[2]	P08253	-0.86	0.78	0.04
Matrix metalloproteinase 14 (MMP-14)[1]	B2R6P3	-0.84	1.12	0.13
Thrombospondin-1 (TSP-1)[1]	P07996	-0.91	1.12	0.10
Collagen I[1]	P02452	0.27	0.60	0.33
Collagen VI[1]	P12109	0.39	0.67	0.22
